# *Cryptococcus neoformans* as a Model for Radioimmunotherapy of Infections

**DOI:** 10.1155/2011/830286

**Published:** 2011-05-24

**Authors:** Ekaterina Dadachova, Arturo Casadevall

**Affiliations:** ^1^Department of Nuclear Medicine, Albert Einstein College of Medicine of Yeshiva University, Bronx, NY 10461, USA; ^2^Department of Microbiology and Immunology, Albert Einstein College of Medicine of Yeshiva University, Bronx, NY 10461, USA; ^3^Department of Medicine, Albert Einstein College of Medicine of Yeshiva University, Bronx, NY 10461, USA

## Abstract

There is an obvious and urgent need for novel approaches to treat infectious diseases. The use of monoclonal antibodies in therapy of infectious diseases is now experiencing renewed interest. During the last 5 years radioimmunotherapy (RIT), a modality previously developed only for cancer treatment, has been successfully adapted for the treatment of experimental fungal, bacterial, and viral infections. As our model organism for studying the efficacy, mechanisms, potential toxicity, and radioresistance to RIT, as well as for comparison of RIT with the existing antimicrobial therapies we have chosen the encapsulated yeast *Cryptococcus neoformans* (CN). The success of RIT approach in laboratory studies provides encouragement for feasibility of therapeutically targeting microbes with labeled antibodies. In addition, the creation of “panantibodies” for RIT which would recognize antigens shared by the whole class of pathogens such as fungi, for example, would facilitate the introduction of RIT into the clinic.

## 1. Introduction

The need for novel approaches to treat infectious diseases at a time of increasing drug resistance and the emergence of new pathogens is obvious and urgent. In recent decades the problem of drug resistance has been compounded by the emergence of many new infectious diseases like HIV. Simultaneously the population of patients in whom current antimicrobial therapies are not effective because of their low immune status is expanding and these include HIV-infected individuals, cancer patients undergoing chemotherapy, and recipients of organ transplants. In addition, there is a threat of biological agents specifically engineered to be lethal even in immunocompetent population.

This situation has renewed interest in using monoclonal antibodies (mAbs) in therapy of infectious diseases [1]. Radioimmunotherapy (RIT) relies on antibodies to deliver cytotoxic alpha- or beta radiation to tumor cells [2]. Radiolabeled mAb Zevalin and Bexxar are FDA approved for untreated, refractory, and recurrent lymphomas. Several years ago we introduced RIT into the realm of infectious diseases, showing prolonged survival in mice systemically infected with CN and treated after infection with radiolabeled mAb specific for CN polysaccharide capsule [3]. During the last 7 years we have successfully adapted RIT for the treatment of experimental fungal, bacterial, and viral infections [4–7]. 

As our model organism for studying the efficacy, mechanisms, potential toxicity, and radioresistance to RIT, as well as for comparison of RIT with the existing antimicrobial therapies we have chosen the encapsulated yeast *Cryptococcus neoformans *(CN). CN has a worldwide distribution and is a major fungal pathogen in immunocompromised hosts responsible for nearly one million serious infections annually and 600,000 deaths [8]. Although the burden of disease is disproportional in individuals with HIV infection, there remains a major risk for cryptococcosis in transplant patients or individuals receiving immunosuppressive drugs, as well as in patients with cancer, cirrhosis, and a variety of other medical conditions. Its major virulence factors are polysaccharide and melanin pigment in the cell wall. Over the past decade, *Cryptococcus gattii* has gained significant public attention as the causative agent of devastating pulmonary and central nervous system infections in immunocompetent individuals principally in the Northwestern USA and Canada. 

Humoral immunity to CN has been extensively studied by Casadevall's laboratory for almost 20 years. Two mAbs generated by his laboratory—18B7 mAb to CN capsular polysaccharide antigen and 6D2 mAb to melanin—have been used in clinical trials: trial of naked 18B7 in patients with cryptococcal meningitis has been completed [9]; and in collaboration with Dadachova 188-Rhenium-labeled 6D2 is currently undergoing trial in patients with metastatic melanoma [10, 11]. CN provides an excellent model for a chronic infection and advantages of the CN system include (1) animal models including those for pulmonary, meningeal, and latent infection; (2) the availability of very well-characterized mAbs to CN that can be developed into RIT agents; (3) the availability of anti-idiotypic reagents that can be used to study the fate of labeled mAbs; (4) well-understood pathogenesis of infection and immune response.

Here we will present the summary of the therapeutic efficacy of RIT of CN, its toxicity and potential for radioresistance, radiobiological mechanisms, and comparison with the standard antifungal therapy and we will outline future perspective for developing RIT into the universal anti-fungal modality in immunocompromised patients.

## 2. Efficacy of RIT of CN

**Figure 1:**
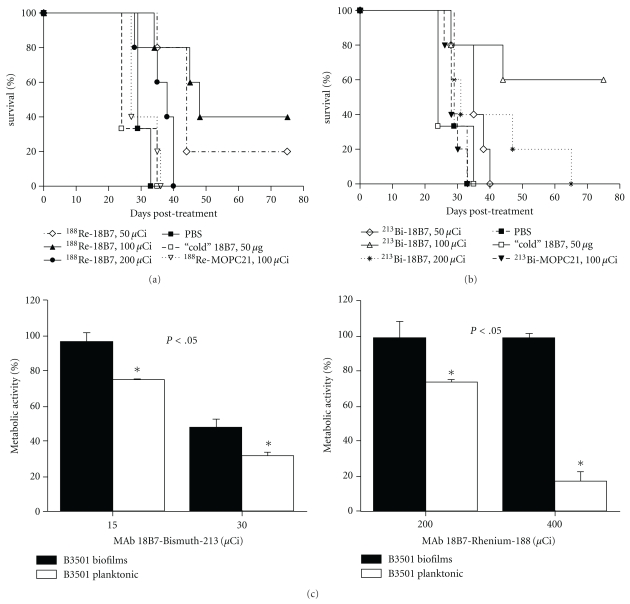
Efficacy of RIT of CN with ^213^Bi- and ^188^Re- labeled mAbs: (a, b) Kaplan-Meier survival curves for A/JCr mice infected IV with 10^5^ *C. neoformans* cells 24 hr prior to treatment with 50–200 *μ*Ci ^188^Re- (a) or ^213^Bi-labeled (b) mAbs. Animals injected with PBS (phosphate buffered saline) or 50 *μ*g “cold” 18B7 served as controls; (c) treatment of CN biofilms in vitro with ^213^Bi- (left panel ) or ^188^Re- (right panel) labeled 18B7 mAb; adapted from [3, 12].

We initially explored the potential efficacy of RIT against a systemic CN infection in partially complement deficient AJ/Cr mice (National Cancer Institute, Frederick, MD). The results discussed below are published in [3]. We radiolabeled CN polysaccharide capsule-specific mAb 18B7 with alpha-particle emitting 213-Bismuth (^213^Bi) or the beta-particle emitting 188-Rhenium (^188^Re). Mice treated with radiolabeled 18B7 mAb lived significantly longer than mice given irrelevant labeled IgG1 or PBS. We used a labeled irrelevant mAb (^213^Bi- or ^188^Re-labeled IgG_1_ MOPC21) to control for the possibility that Fc receptor binding by the radiolabeled IgG to phagocytes at the site of infection might result in nonspecific killing of CN cells. Remarkably, 60% of mice in 100 *μ*Ci ^213^Bi group were alive on day 75 after therapy (*P* < .05). In the ^188^Re group, 40% and 20% of animals were alive after treatment with 100 (*P* < .005) and 50 *μ*Ci (*P* < .05) ^188^Re-18B7, respectively, while mice in control groups succumbed to infection on days 35–40 (Figures 1(a) and 1(b)). Mice infected with CN and given RIT had significantly reduced fungal burden in lungs and brains 48 h after treatment when compared to control groups. While there was no difference in the reduction of the fungal burden in the lungs between the groups that received 50 and 100 *μ*Ci ^188^Re-18B7, treatment with 200 *μ*Ci ^188^Re-18B7 significantly lowered lung CFUs relative to the lower activities (*P* < .05). Hence, administration of a radiolabeled antibody to CN polysaccharide prolonged survival and reduced organ fungal burden in infected mice.

When the RIT dose dependence was investigated, survival of A/JCr mice was dose dependent for both ^213^Bi and ^188^Re radioisotopes: while 50 *μ*Ci ^213^Bi-18B7 produced no therapeutic effect, both the 100 and 200 *μ*Ci doses prolonged animal survival [3]. Interestingly, the 200 *μ*Ci ^213^Bi-18B7 dose was less efficient, possibly because it may have approached the MTA (maximum tolerated activity) for this particular combination of antibody and radioisotope. 

Later we evaluated the efficacy of RIT against fungal biofilms. The results discussed below are published in [12]. The use of indwelling medical devices—pacemakers, prosthetic joints, and catheters—is rapidly growing and is often complicated by infections with biofilm-forming microbes that are resistant to antimicrobial agents and host defense mechanisms. We investigated the use of polysaccharide-specific mAbs as delivery vehicles for targeting *C. neoformans* biofilms with ^213^Bi. ^213^Bi-18B7 mAb (IgG1) penetrated cryptococcal biofilms, as shown by confocal microscopy and caused a 50% reduction in biofilm metabolic activity (Figure 1(c) left panel). In contrast, when the IgM mAb 13F1 labeled with ^213^Bi was used—there was no penetration of the fungal biofilm and no damage. Unlabeled 18B7, ^213^Bi-labeled nonspecific mAbs, and gamma and beta types of radiation (Figure 1(c) right panel) did not have an effect on biofilms. The lack of efficacy of gamma and beta radiation probably reflects the radioprotective properties of polysaccharide biofilm matrix. Our results indicate that CN biofilms are susceptible to treatment with antibody-targeted alpha radiation, suggesting that RIT could provide a novel option for the prevention or treatment of microbial biofilms on indwelling medical devices.

## 3. Toxicity of RIT of CN

**Figure 2:**
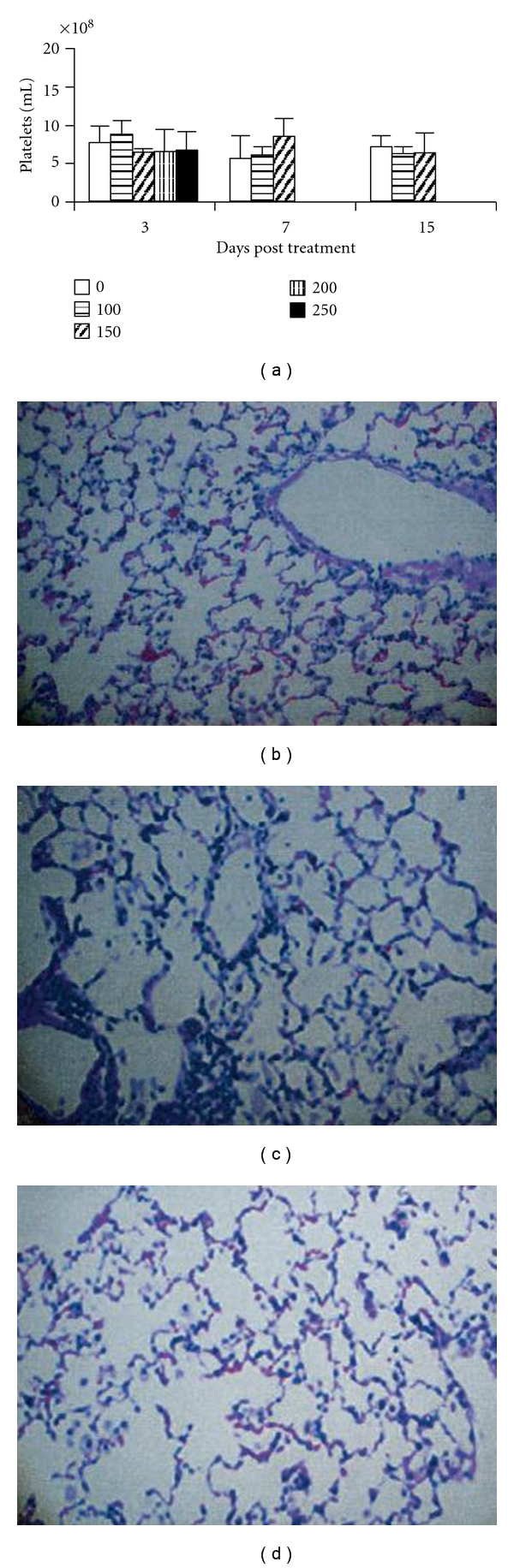
Toxicity of RIT in mice with CN infections. (a) Platelet counts in RIT-treated mice. CN-infected A/JCr mice received various doses of ^213^Bi-18B7. A “0” indicates infected nontreated mice. Mice treated with 200 and 250 *μ*Ci ^213^Bi-18B7 died by day 7 posttreatment; (b–d) micrographs of hematoxylin-and-eosin-stained lungs from BALB/c mice infected IT with CN and treated with radiolabeled mAbs. Mice were sacrificed 5 months after RIT: (b) infected control group (no RIT); (c) 200 *μ*Ci ^213^Bi-18B7; (d) 200 *μ*Ci ^188^Re-18B7; adapted from [13].

While it was known from the cancer RIT data that the platelet counts nadir usually occurred 1 week after radiolabeled antibody administration to tumor-bearing mice [14, 15]—there was no information about possible toxic effects of RIT in infected animals. In our studies of RIT for murine cryptococcosis we evaluated the hematological toxicity of radiolabeled antibodies in mice by platelet counts [13]. In AJ/Cr mice systemically infected with CN no changes in platelet counts were observed for the doses of up to 150 *μ*Ci ^213^Bi- or ^188^Re-labeled mAbs (Figure 2(a)) attesting to the lack of the hematologic toxicity in this range while mice given 200 and 250 *μ*Ci died by day 7 posttreatment [13]. 

We also considered the possibility that RIT of CN infection may promote lung fibrosis in treated animals. Lungs are the target organ for CN infection and it is known from cancer field that lungs can develop fibrosis several months after treatment with external beam radiation therapy [16]. To evaluate this potential complication we used a pulmonary model of CN where mice are infected intratracheally (IT). In this model, CN is mostly localized to the lungs on day 5 after infection, and as a result up to 10% of the injected dose/g was found in the lungs at 24 h after treatment with radiolabeled MAbs, versus 1.5% of the injected dose/g in the lungs of non-infected mice [3]. The results described below are published in [13]. BALB/c mice were infected IT with 10^6^ CN cells, and on day 5 after infection they were treated with 50–200 *μ*Ci ^213^Bi- or ^188^Re-labeled mAbs or left untreated. All mice were subsequently maintained on fluconazole to control infection (10 mg/kg in their drinking water). After 5 months, the mice were sacrificed, and their lungs were removed, fixed with buffered formalin, sectioned, stained with hematoxylin and eosin, and analyzed histologically. There was no evidence of radiation fibrosis in the lungs of radiation-treated mice (Figures 2(c) and 2(d)) compared to control animals (Figure 2(b)). This lack of hematological and pulmonary toxicity can be explained by the very specific targeting of radiolabeled antibodies to the microbes/infected cells. In fact, one of the advantages of using RIT against infections as opposed to cancer is that, in contrast to tumor cells, cells expressing microbial antigens are antigenically very different from host tissues and thus provide the potential for exquisite specificity and low cross-reactivity. It should also be noted that in all our studies the radiolabeled mAbs were administered ip, and ip administration of the radiolabeled mAbs was reported to be better tolerated than iv route [17]. In addition, when using a radioactive therapy in patients there is always a concern of long-term effects such as neoplasms arising from radiation-induced mutations. However, this risk should be extremely low after short-term exposure and would likely be outweighed by the benefits of treating or preventing infections. Nevertheless, the application of RIT to infectious diseases will require optimization of the dose to ascertain and minimize toxic effects. 

## 4. RIT and Radiation Resistance

**Figure 3:**
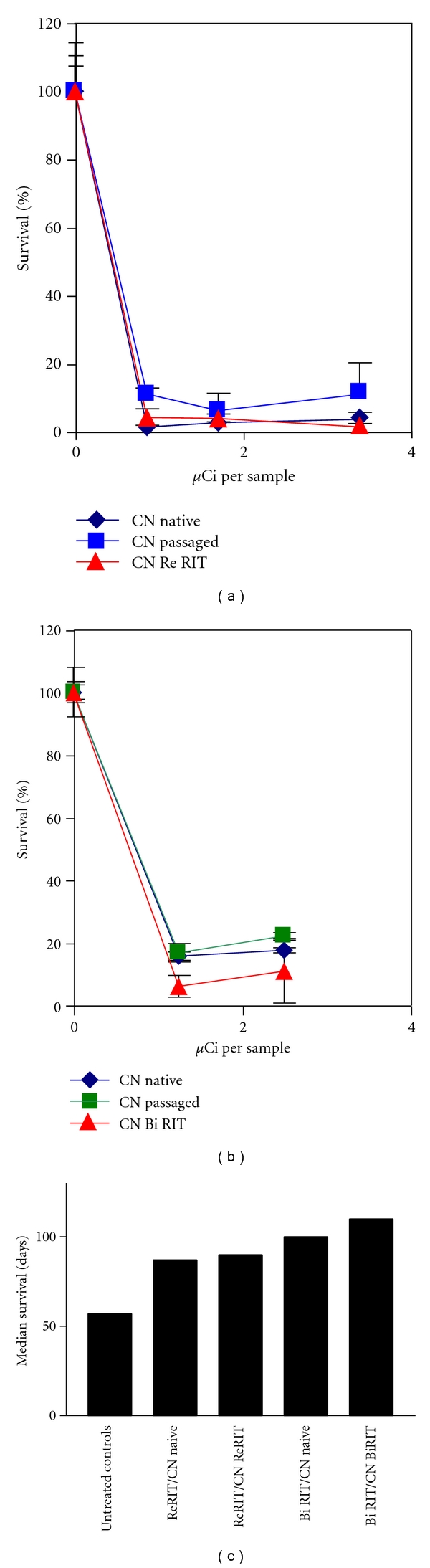
Investigation of possible resistance to RIT of CN in vivo and in vitro: (a) in vitro killing of CN cells with ^188^Re-18B7 mAb. Each sample contained 10^5^ fungal cells; (b) in vitro killing of CN cells with ^213^Bi-18B7 mAb. Each sample contained 10^5^ fungal cells; (c) median survival of AJ/Cr mice infected IV with 5 × 10^4^ CN and treated 24 hrs later with 150 *μ*Ci ^188^Re-18B7 or 125 *μ*Ci ^213^Bi-18B7 mAb. CN_naive_: cells from ATCC; CN_passaged_: cells recovered from untreated AJ/Cr mice; CN_Re RIT_: cells recovered from mice treated with ^188^Re-18B7 mAb; CN_Bi RIT_: cells recovered from mice treated with ^213^Bi-18B7 mAb; Re RIT/ CN_naive_: mice infected with CN_naive_ and treated with ^188^Re-18B7; Bi RIT/ CN_naive_: mice infected with CN_naive_ and treated with ^213^Bi-18B7; Re RIT/ CN_Re RIT_: mice infected with CN_Re RIT_ and treated with ^188^Re-18B7; Bi RIT/ CN_Bi RIT_: mice infected with CN_Bi RIT_ and treated with ^213^Bi-18B7; adapted from [18].

The emergence of radiation-resistant CN cells would be a concern for multiple RIT administrations, and therapeutic outcome. Thus, we evaluated susceptibility of CN cells isolated from RIT-treated mice to RIT in vitro. The results discussed below are published in [18]. To generate RIT-treated CN cells, AJ/Cr mice were infected IV with 5 × 10^4^ cells and 24 hrs later treated with either 150 *μ*Ci ^188^Re-18B7 or 125 *μ*Ci ^213^Bi-18B7 or left untreated. The surviving mice were sacrificed, their lungs homogenized and plated on SAB agar; isolated colonies were grown overnight in SAB broth. To assess radiosensitivity of the cells in vitro, cells from ATCC (CN_naive_), recovered from untreated AJ/Cr mice (CN_passaged_) and recovered from mice given ^188^Re-18B7 mAb (CN_Re RIT_) or ^213^Bi-18B7 mAb (CN_Bi RIT_) were treated with ^188^Re- or ^213^Bi-18B7 mAb as in [3]. Naive, passaged, or RIT pretreated cells were equally radiosensitive to both ^188^Re and ^213^Bi attesting to the absence of in vitro radioresistance of RIT-pretreated cells (Figures 3(a) and 3(b)). 

To evaluate the possibility that RIT might select for CN cells resistant to radiation in vivo, we infected AJ/Cr mice with CN_Re-RIT_, CN_Bi-RIT_ and CN_naive_. Infected mice were treated with 150 *μ*Ci ^188^Re-18B7 or 125 *μ*Ci ^213^Bi-18B7 24 hrs after iv infection, then monitored for survival and weight loss. Lethality in mice infected with CN_Re-RIT_ or CN_Bi-RIT_ was the same as in mice infected with CN_naive_ (*P* > .05) (Figure 3(c)). Survival of mice treated with ^213^Bi-18B7 mAb was longer (*P* = .04) than with ^188^Re-18B7 (Figure 3(c)), probably due to the higher killing power of alpha particles from ^213^Bi, compared to electrons from ^188^Re. Overall, the treatment of CN with particulate radiation leads to loss of the ability of the cells to replicate [3, 19], which would explain the absence of radiation-resistant phenotypes after RIT. The residual cells which replicate after RIT most likely were protected from radiolabeled antibodies by a biofilm, an abscess, or a host cell.

## 5. Radiobiological Mechanisms of RIT of CN

Given that RIT of infectious diseases is a relatively young field, the mechanisms by which RIT is effective are uncertain. Even in oncology where the antineoplastic effects of RIT have been investigated for more than 25 years the cytotoxic mechanisms are still debated. The major radiobiological mechanisms of cancer RIT are considered to be “direct hit” (when a cell is killed by radiation emanating from the same cell) and “cross-fire” effects (when a cell is killed by radiation emanating from a distant cell), both of which can promote apoptosis and cell cycle redistribution [20]. We investigated the radiofungicidal effects of external gamma radiation and ^213^Bi- or ^188^Re-labeled mAbs on CN cells by evaluating the effect of radiofungicidal doses on cell membrane permeability, induction of apoptosis, and cellular metabolism. The results discussed below are published in [19].

**Figure 4:**
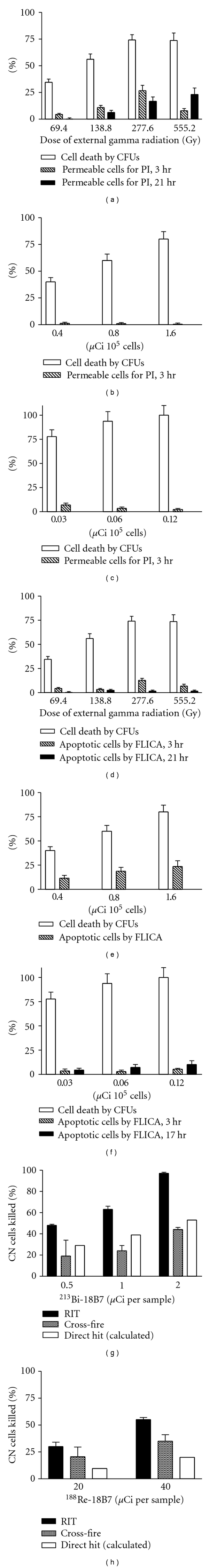
Contribution of different radiobiological effects to RIT of CN with ^213^Bi-18B7 and ^188^Re-18B7 mAbs. (a–c) show CFUs and PI permeability for: (a) external gamma radiation; (b) ^188^Re-18B7; (c) ^213^Bi-18B7. (d–f) show CFUs and apoptosis levels by FLICA: (d) external gamma radiation; (e) ^188^Re-18B7; (f) ^213^Bi-18B7. (g–h) show contribution of “cross-fire” and “direct hit” towards killing of CN cells: (g) “cross-fire” and “direct hit” for ^213^Bi-18B7; (h) “cross-fire” and “direct hit” for ^188^Re-18B7. The contribution of “direct hit” towards cell killing was calculated by subtracting percentage of cells killed by “cross-fire” from percentage of cells killed by RIT; adapted from[19, 21].

An increased membrane permeability to the dye propidium iodide (PI) is considered to be a marker of cell death since viable cells with intact membranes are able to exclude the dye. Internalized PI binds to nucleic acids and undergoes a large increase in fluorescence [22]. PI staining correlates with loss of colony forming units (CFUs) in a variety of microorganisms including CN treated with antifungal agents [23]. The permeability increased with time between 1 and 3 hr following gamma irradiation, indicating that it was probably secondary to cell death, not a cause of death (Figure 4(a)). It seems likely that the cells in this 20% of the population are metabolically “dead” and unable to maintain membrane integrity. Cells stained 3 hr after irradiation showed dose-dependent PI staining up to 300 Gy (25% PI positive), with a decrease to 10% PI positive at the highest dose (Figure 4(a)). This observation suggests that membrane damage is not the primary lethal event, as 80% of the cells had lost the ability to replicate at these doses. The decrease in PI positive cells at the highest dose may be due to radioprotective effects from the shed capsule [24]. Treatment of CN with ^188^Re-18B7 did not make the cells PI permeable (Figure 4(b)). Treatment with ^213^Bi-18B7 mAb led to about 7% of the cells becoming PI permeable, at a dose that caused 80% loss of CFUs (Figure 4(c)). 

Fungal cells undergo apoptosis or programmed cell death [25]. In the same paper [19], we investigated whether radiation increased levels of fungal caspase, as measured by FLICA (fluorochrome labeled inhibitor of caspase) binding—a membrane permeable substrate that binds to caspases induced during early apoptosis. Earlier, we validated this technique for use with CN by comparing the FLICA results with those obtained using APO-BrdU TUNEL apoptosis detection kit [21]. Gamma-irradiated cells were about 10% FLICA positive at 3 hr (Figure 4(d)) while 20 and 5% of CN cells exposed to ^188^Re-18B7 or ^213^Bi-18B7 mAbs, respectively, became FLICA positive (Figures 4(e) and 4(f)). The number of FLICA positive ^213^Bi-18B7 mAb-treated cells staining was higher at 17 hrs than at 3 hrs, indicating an ongoing process of apoptosis induction. Apoptosis is a dynamic process, and cells pass through several stages, not staying at any one stage for a long time. The decrease seen at 21 hrs for the gamma-radiation treated cells may indicate that at that time the cells have finished the stage of apoptosis during which the caspases are available to bind the fluorescent inhibitors. This is in contrast to the increase with time observed for ^213^Bi-18B7 mAb treated cells and may reflect a difference in pathways of cell death induced by the different forms of radioactivity. We concluded that gamma, beta, and alpha radiation affected cells via different pathways. Gamma radiation had more effect on the cell membrane than ^213^Bi-18B7 or ^188^Re-18B7. All forms of radiation stimulated apoptosis-like cell death with gamma radiation and ^188^Re-18B7 mAb having more pronounced effect than ^213^Bi-18B7 mAb. ^213^Bi-18B7 mAb delivered “directly” decreased the metabolic activity of fungal cells, while the other forms of radiation did not. Clonogenic survival proved to be the most practical measure of assessing RIT efficacy, by virtue of reflecting a combination of multiple mechanisms leading to fungal cell death. Cells which are alive after RIT treatment, but not replicating, may or may not contribute to the disease.

To elucidate the contribution of “direct hit” and “cross-fire” effects to RIT of CN we compared the fungicidal activity of a mAb radiolabeled with ^213^Bi or ^188^Re—isotopes with different emission ranges in tissue −50–80 *μ*m for ^213^Bi versus 10 mm for ^188^Re. In cancer RIT, ^213^Bi is assumed to kill by “direct hit”, while ^188^Re, through “cross-fire”. In principle, every cell with bound radiolabeled mAb molecules can be killed by a “direct hit” and simultaneously serve as a source of “cross-fire” radiation. By measuring the killing of the cells in RIT and in “cross-fire” experiments, we can calculate contribution of “direct hit” towards cell killing by subtracting percentage of cells killed by “cross-fire” from percentage of cells killed by RIT. The results discussed below are published in [21]. To observe “cross-fire” we had to ensure that the cells that served as the sources of “cross-fire” radiation could not be killed themselves by “direct hit”. Consequently, we used heat killed CN cells as the sources of “cross-fire” radiation. Experiments with ^213^Bi-18B7 showed that although most fungal cells were killed by “direct hit”, “cross-fire” effect also contributed to the fungicidal effect of RIT (Figure 4(g)). No killing of CN cells by unlabeled mAb 18B7 was observed. For ^188^Re-18B7 “cross-fire” effect was responsible for most of CN killing (Figure 4(h)). This system permits experiments to elucidate precise mechanisms of cell killing in RIT that have not been performed either for microbial or cancer cells. In RIT targeting of cancer cells the antibody is often internalized after binding, adding significant complexity to the experiment. One of the advantages of the CN system is that the capsule is outside the cell wall and that antibody is not internalized, thus allowing exploration of this fundamental problem in radiobiology. One minor limitation of this system is that the antibody could be internalized by phagocytes that ingest the antibody-labeled CN. Knowledge of the radiobiological mechanisms of RIT will allow creation of more effective protocols for RIT of opportunistic fungal infections.

## 6. Comparison of RIT of CN with Standard Antifungal Treatment

**Figure 5:**
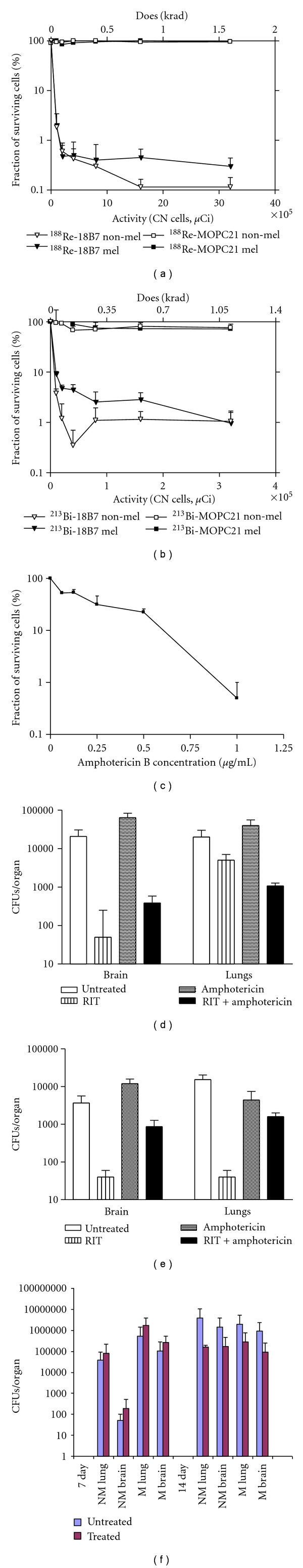
Comparison of RIT and amphotericin efficacy towards nonmelanized and melanized CN in vitro and in vivo. (a–c) In vitro killing and dosimetry of melanized and nonmelanized CN cells treated with: (a) ^188^Re-labeled 8B7 and control isotype-matching MOPC21 mAbs; (b) ^213^Bi-labeled 18B7 and control MOPC21 mAbs; (c) amphotericin B; mel: melanized CN cells; non-mel: nonmelanized CN cells; (d, e) CFUs in the lungs and brains of mice infected with nonmelanized or melanized CN. AJ/Cr mice were infected IV with 3 × 10^5^ CN cells and 24 hr later either given 100 *μ*Ci ^213^Bi-18B7 RIT or amphotericin B at 1 *μ*g/g body weight on Days 1, 2, and 3 after infection or combined treatment or left untreated: (d) nonmelanized CN; (e) melanized CN. Detection limit of the method was 50 CFUs. No CFUs were found in the brains and lungs of mice infected with melanized CN cells and treated with RIT which are presented in the graph as 40 CFUs/organ; (f) CFUs in the brain and lungs of mice infected with 3 × 10^5^ melanized (M) or nonmelanized (NM) CN cells and treated with amphotericin B at 1 *μ*g/g body weight for 14 days. Mice were sacrificed at days 7 and 14 posttreatment; adapted from [26].

As an important step towards bringing RIT of fungal diseases into the clinic, we compared the efficacy of RIT versus amphotericin against systemic experimental CN infection. The results discussed below are published in [26]. We hypothesized that 18B7 mAb radiolabeled with ^213^Bi or with ^188^Re would be able to kill both melanized and nonmelanized CN cells in vivo better than standard antifungal therapy. We also investigated whether the combination of RIT and amphotericin treatment produced different results from either therapy alone. For this melanized and nonmelanized 24067 CN cells were incubated with increasing activities of ^188^Re- and ^213^Bi-18B7 mAb. Incubation of melanized and nonmelanized cells with ^188^Re- or ^213^Bi-18B7 mAb killed 90% of the cells and delivered cellular radiation doses of 0.1 krad for ^188^Re-18B7 and 0.04 krad for ^213^Bi-18B7 (Figures 5(a) and 5(b)). ^213^Bi or ^188^Re conjugated to the irrelevant isotype-matching antibody MOPC killed neither type of cell (Figures 5(a) and 5(b)). The difference in susceptibility of melanized and nonmelanized cells to antibody-delivered radiation became obvious when we attempted to achieve 99.9% elimination of cells. Sixteen *μ*Ci (0.8 krad dose) of ^188^Re-18B7 mAb eliminated 99.9% of nonmelanized cells, while that degree of cell killing was not achieved for melanized cells in the investigated range of activity. ^213^Bi-18B7 mAb killed 99.7% of nonmelanized cells with 0.4 *μ*Ci (0.17 krad dose) but again that level of cell killing was not observed for melanized cells. As approximately 10 times less ^213^Bi than ^188^Re radioactivity was required to eliminate the bulk of either melanized or nonmelanized cells we selected ^213^Bi-mAbs for in vivo comparison with amphotericin. One *μ*g/mL amphotericin reduced CN CFUs by more than two log units (Figure 5(c)). Considering published MIC for melanized 24067 CN being higher than for nonmelanized; we selected a dose of 1 *μ*g/gram of mouse body weight (~17*μ*g/mouse), allowing a transient blood concentration of 8.5 *μ*g/mL, for in vivo experiments.

Subsequently we compared the efficacy of RIT alone to that of amphotericin and combined therapy in vivo. AJCr mice were infected iv with 3 × 10^5^ melanized or nonmelanized CN cells. One day after infection mice were divided into groups of 5 that were either untreated; or given ip 100 *μ*Ci ^213^Bi-18B7; or treated at 24, 48, and 72 h with amphotericin as deoxycholate at 1 *μ*g/g body weight; or received both treatments. Mice were monitored for survival for 60 days. Analysis of lungs and brains at 60 days after infection showed that amphotericin did not significantly decrease CFUs in the lungs and the brains in either nonmelanized (Figure 5(d)) or melanized CN groups (Figure 5(e)) (*P* > .05). RIT had significantly decreased fungal burdens compared to untreated or amphotericin-treated mice (*P* ≪ .05). In fact, RIT-treated nonmelanized CN group almost completely cleared fungus from the brain (the lower limit of detection was 50 CFUs), while RIT-treated melanized CN group almost completely cleared the infection from both brain and lungs. 

Our most important observation is that RIT was more effective in reducing fungal burden in lungs and brains than amphotericin at a high dose of 1 *μ*g/g, with most RIT-treated mice almost completely clearing the infection. The inability of amphotericin to reduce the fungal burden in the organs of partially complement deficient AJCr mice after 3 days of treatment was explained by the follow-up study with a trend towards reduction of CFUs in brains and lungs manifesting itself only on the 14th day of treatment (Figure 5(f)). These observations are in concert with literature showing that even in intact robust mice as CD-1 or Balb/c amphotericin as deoxycholate was also only able to produce 1–1.5 log reduction in CFUs and all mice died around day 24 [27, 28]. It is also in concert with the data from clinical studies showing that a short course of amphotericin does not sterilize cerebrospinal fluid or blood, and that the rate of sterilization correlates with survival [29]. Our observation underlines the advantages of RIT which produces microbicidal effects in vivo just after one injection when compared to prolonged treatment with amphotericin. When combined RIT and amphotericin treatment was used—a complex picture emerged depending on the melanization status of infection. Combination treatment was more effective than amphotericin alone for both nonmelanized and melanized CN groups. In melanized CN group the combination treatment was less effective than RIT which could be due to inflammation and renal toxicities associated with amphotericin at this dose in mice. Interestingly, for nonmelanized CN the combination treatment did produce some synergy in reducing CFUs in the lungs. It is possible to suggest that if RIT is administered much later during the course of treatment with amphotericin; some synergistic effects could be observed.

## 7. Conclusions

The success of RIT of CN in laboratory studies combined with earlier nuclear medicine experience on preclinical and clinical studies showing the utility of radiolabeled organism-specific antibodies for imaging of infections (reviewed in [30]) provides encouragement for feasibility of therapeutically targeting microbes with labeled antibodies. In fact, the ability of a specific antibody to localize to a site of infection indicates the feasibility of using the antibody-antigen interaction to deliver microbicidal radiation to sites of infection, which in turn provides strong support for the potential usefulness of this technique as a broad antimicrobial strategy. As microbial cells are foreign to the human body; they contain antigens that are not expressed by human tissues and this provide a major contrast to cancer RIT since tumor-associated antigens are also expressed on normal tissues. Consequently, the theoretical therapeutic index of RIT for microbial diseases should be significantly higher than for neoplastic diseases. This exquisite specificity promises exclusivity of targeting which should translate into high efficacy of treatment and low toxicity. It might be possible to create a so-called “pan-antibody” which would recognize an antigen shared by a particular class of human pathogens such as fungi, for example. Example of such “panantibodies” is a mAb 6D2 initially developed against fungal melanin which also binds to synthetic, invertebrate (cuttlefish), murine and human melanin [10]; mAbs to heat shock protein 60 (HSP60) [31] and beta-glucans [32] which bind to all major human pathogenic fungi. The experiments on developing RIT with such panantibodies are currently ongoing in our laboratories (Bryan et al. unpublished observations). The availability of such antibodies would eliminate the necessity of having antibodies specific for each particular microorganism and would enormously enhance the development of RIT of infectious diseases.
